# 

**Published:** 1880-02

**Authors:** 


### BOOKS AND PAMPHLETS RECEIVED.

My Struggle with a Tape-worm. By G. B. Pratt, M.D., Elkhart, Ind., late Resident Physician of the King's County Hospital, Brooklyn.

Dental Hints for Country Medical Practitioners. By G. Newkirk, M.D., Wenona, Ill.

Valedictory Address to the Graduating Class of the Medical Department of the University of California. By W. F. McNutt, M.D., L.R.C.P., Ed., etc., etc., Professor of Principles and Practice of Medicine, Med. Dep. University of California.

A Manual of the Practice of Surgery. By W. Fairlie Clarke, M.A. and M.B. (Oxon), F.R.C.S., Assistant Surgeon to the Charing Cross Hospital. From the last London edition. Revised and edited, with additions, by an American Surgeon. New York: William Wood & Co. 1879.

On the Use of Water in the Treatment of Diseases of the Skin. By L. Duncan Bulkley, A.M., M.D., Physician to the Skin Dept., Demilt Dispensary, New York, etc., etc.

A Contribution to the Hæmatinic Properties of Dialyzed Iron. By Robert Amory, M.D., of Longwood, Mass.

Pay Hospitals and Paying Wards throughout the World. Facts in Support of a Re-arrangement of the English System of Medical Relief. By Henry C. Burdett, late General Supt. of the Queen's Hospital, Birmingham. London: J. & A. Churchill, New Burlington St., W. 1879.

Responsibility Restricted by Insane Delusion. By T. L. Wright, M.D., Bellefontaine, O.

A Clinical Inquiry into the Diagnostic Significance of Absent Patellar Tendon Reflex. By C. H. Hughes, M.D., late Superintendent and Physician to the Missouri State Lunatic Asylum, Fulton, etc., etc.

Saint Bartholomew's Hospital Reports. Edited by W. S. Church, M.D., and Alfred Willett, F.R.C.S. Vol. XV. London: Smith, Elder & Co., 15 Waterloo place; 1879.

---

DR. J. ADAMS ALLEN, President of Rush Medical College, has been compelled, in consequence of the state of his health, to take a respite from the labor incidental to his practice as a physician and his duties as a college professor. He sailed for Europe January 17th, in the company of his wife, and intends to visit Egypt first, passing up as far as the Nile cataracts. He expects to return in the fall of this year, and has carried with him the best wishes of his many friends for the complete restoration of his failing health. Prior to his bidding good-bye to the members of his class at the college, he was presented by them with a costly and elegantly engraved gold watch, as a souvenir of their affectionate regard. When he proceeded to take the train at the station, he found there several hundreds of the college students who loudly cheered him as he set out upon his long journey.

### ANNOUNCEMENTS FOR THE MONTH.

### SOCIETY MEETINGS.

Chicago Medical Society—Mondays, Feb. 2 and 16.

West Chicago Medical Society—Mondays, Feb. 9 and 23.

### CLINICS.

MONDAY.

Eye and Ear Infirmary—2 p. m., Ophthalmological, by Prof. Holmes; 3 p. m., Otological, by Prof. Jones.

Mercy Hospital—2 p. m., Surgical, by Prof. Andrews.

Rush Medical College—2 p. m., Dermatological and Venereal, by Prof. Hyde; 3 p. m., Medical, by Dr. Bridge.

Woman's Medical College—3 p. m., Diseases of the Chest, Prof. Ingals.

TUESDAY.

Cook County Hospital—2 to 4 p. m., Medical and Surgical Clinics.

Mercy Hospital—2 p. m., Medical, by Prof. Hollister.

WEDNESDAY.

Chicago Medical College—2 p. m., Eye and Ear, by Prof. Jones.

Rush Medical College—3:30 to 4:30 p. m., Diseases of the Chest, by Dr. E. Fletcher Ingals.

THURSDAY.

Chicago Medical College—2 p. m., Gynæcological, by Prof. Jenks.

Rush Medical College—3 p. m., Diseases of the Nervous System, by Prof. Lyman.

Eye and Ear Infirmary—2 p. m., Ophthalmological, by Dr. Hotz.

Woman's Medical College—3 p. m., Surgical, by Prof. Owens.

FRIDAY.

Cook County Hospital—2 to 4 p. m., Medical and Surgical Clinics.

Mercy Hospital—2 p. m., Medical, by Prof. Davis.

SATURDAY.

Rush Medical College—2 p. m., Surgical, by Prof. Gunn.

Chicago Medical College—2 p. m., Surgical, by Prof. Isham; 3 p. m., Neurological, by Prof. Jewell.

Woman's Medical College—11 a. m., Ophthalmological, by Prof. Montgomery; 2 p. m., Gynæcological, by Prof. Fitch.

Daily Clinics, from 2 to 4 p. m., at the Central Free Dispensary, and at the South Side Dispensary.

